# Assessing the Capability of Large Language Model Chatbots in Generating Plain Language Summaries

**DOI:** 10.7759/cureus.80976

**Published:** 2025-03-21

**Authors:** Himel Mondal, Gaurav Gupta, Pradosh Kumar Sarangi, Shreya Sharma, Pritam K Choudhary, Ayesha Juhi, Anita Kumari, Shaikat Mondal

**Affiliations:** 1 Physiology, All India Institute of Medical Sciences, Deoghar, IND; 2 Pediatrics, All India Institute of Medical Sciences, Guwahati, IND; 3 Radiodiagnosis, All India Institute of Medical Sciences, Deoghar, IND; 4 Neuromodulation Laboratory/Physiology, All India Institute of Medical Sciences, Deoghar, IND; 5 Physiology, Raiganj Government Medical College and Hospital, Raiganj, IND

**Keywords:** artificial intelligence, chatbot, chatgpt, claude, large language model, layman summary, manuscript preparation, medical writing, plain language summary, scientific writing

## Abstract

Background

Plain language summaries (PLSs) make scientific research accessible to a broad non-expert audience. However, crafting effective PLS can be challenging, particularly for non-native English-speaking researchers. Large language model (LLM) chatbots have the potential to assist in generating summaries, but their effectiveness compared to human-generated PLS remains underexplored.

Methods

This cross-sectional study compared 30 human-written PLS with LLM chatbot (viz., ChatGPT (OpenAI, San Francisco, CA), Claude (Anthropic, San Francisco, CA), Copilot (Microsoft Corp., Washington, DC), Gemini (Google, Mountain View, CA), Meta AI (Meta, Menlo Park, CA), and Perplexity (Perplexity AI, Inc., San Francisco, CA)) generated PLS. The readability of the PLS was checked by the Flesch reading (FR) ease score, and understandability was checked by the Flesch-Kincaid (FK) grade level. Three authors rated the text on seven-item predefined criteria, and their average score was used to compare the quality of the PLS.

Results

In comparison to human-written PLS, chatbots could generate PLS with lower FK grade levels (p-value < 0.0001) and except Copilot, all others had higher FR ease scores. The overall score of human-written PLS was 8.89±0.26. Although there was statistically significant variance among the scores (F = 7.16, p-value = 0.0012), in the post-hoc test, there was no difference between human-generated and individual chatbots-generated PLS (ChatGPT 8.8±0.34, Claude 8.89±0.33, Copilot 8.69±0.4, Gemini 8.56±0.56, Meta AI 8.98±0.23, and Perplexity 8.8±0.3).

Conclusion

LLM chatbots can generate PLS with better readability and a person with a lower grade of education can understand it. The PLS are of similar quality to those written by human authors. Hence, authors can generate PLS from LLM chatbots and it is particularly beneficial for researchers in developing countries. While LLM chatbots improve readability, they may introduce minor inaccuracies also. Hence, PLS generated by LLM should always checked for accuracy and relevancy.

## Introduction

The plain language summary (PLS) is an adjunct component of scientific communication, designed to present research findings in a clear, concise, and accessible manner for a broad audience, including policymakers, practitioners, patients, and the general public [1,2]. By translating complex scientific concepts into simple language, PLS bridges the gap between the scientific community and laypersons [3]. Its importance has been increasingly recognized, particularly in fields where public awareness and engagement are crucial, such as healthcare, environmental science, and public policy [4].

Crafting an effective PLS poses numerous challenges for researchers. This task requires the ability to communicate complex ideas straightforwardly [5]. These challenges are exacerbated for non-native English speakers [6]. In developing countries, where resources and training opportunities are often limited, these difficulties can hinder the dissemination of research findings and limit the impact of valuable scientific contributions on a global scale [7].

Large language model (LLM) chatbots offer a potential solution to these challenges. These tools are capable of understanding and generating human-like text, making them suitable to assist researchers in creating PLS [8,9]. LLM chatbots can tailor the readability and tone of summaries according to the target audience [10].

A study by Van Veen et al. showed that medical science-tuned LLM can perform better than humans in text summarization [11]. In contrast, Tang et al. found that general-purpose LLM like ChatGPT (Open AI, San Francisco, CA) can summarize text but can generate inconsistent and overly convincing summaries [12]. In contrast, Ovelman et al. reported that an LLM - Claude 2 can generate PLS with accuracy and with minor errors [13]. As the field of LLM is expanding and there are several freely accessible chatbots, it is necessary to explore their comparative capability in generating PLS.

This study aims to assess the readability, accuracy, and quality of LLM-generated PLS compared to human-written ones. To achieve this, we conducted a cross-sectional study comparing chatbot-generated and human-generated PLS. The findings would have significant implications for researchers, particularly in developing countries, by offering a practical tool to improve the clarity and impact of their work.

## Materials and methods

Type and settings

This was a cross-sectional study conducted with public domain (freely accessible) data from various websites. It was designed as a comparative analysis of the readability of PLS generated by LLM chatbots versus those written by human authors. The study was conducted from July 1 to July 30, 2024. As this study only audited public domain data, and there are no human or animal research participants, this study does not require ethics committee approval.

Data source

**Figure 1:**
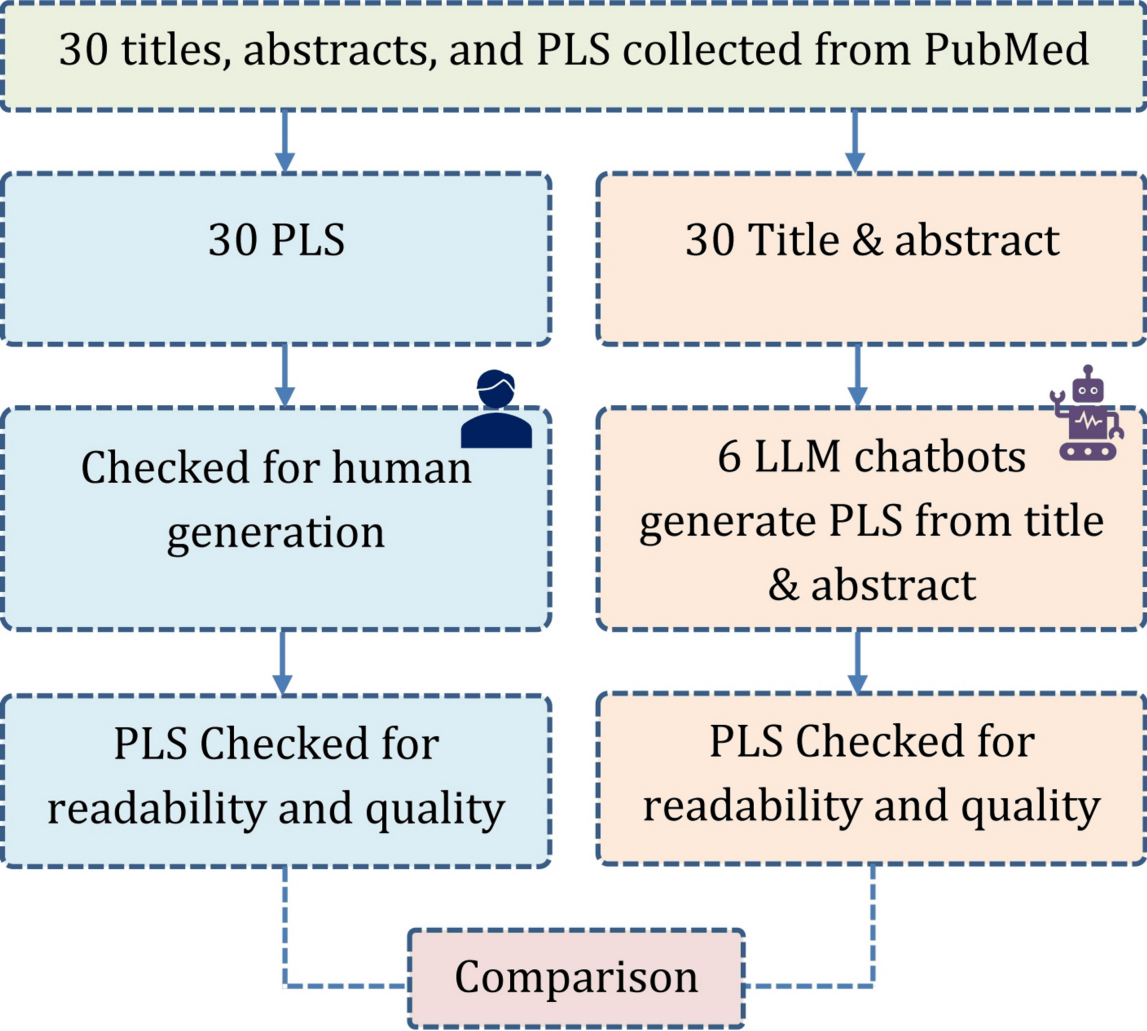
Overall study process in brief PLS: Plain language summary, LLM: Large language summary

We searched “plain language summary” in PubMed to identify relevant articles published with PLS between January 1, 2000 to December 31, 2021. The cutoff year 2021 was selected as the prominent LLM chatbot - ChatGPT was introduced in 2022 [14]. Hence, it is assumed that the authors wrote the PLS themselves without the help of the generative AI. From the PubMed search results, titles, abstracts, and their corresponding PLS were extracted from a total of 30 articles, ensuring a maximum of four articles from any single journal to maintain diversity. We included all the article types (e.g., trial, observational study, review, case report) that appeared in the search result, till the completion of 30 items. These human-generated PLS served as a benchmark for comparison with the LLM-generated summaries. To confirm or reinforce our assumption of human generation, we used an online tool available at https://quillbot.com/ai-content-detector, and all 30 PLS collected were found to be written by humans. The overall study process is briefly presented in Figure 1.

LLM selection

Based on a comprehensive review of current literature and personal experience, three authors (HM, AJ, and SM) made a list of five chatbots and a consensus was reached among the authors to finally select six LLM chatbots for this study. There is an Indian company-backed chatbot called Krutrim. It could not be included as it does not allow a large volume of text as an input command. The selection criteria focused on the free access or at least some amount of free access per day, accessible from the country, previously used in any study, or personal experience of using it. The chatbots were - ChatGPT-4o (OpenAI), Claude Sonnet (Anthropic, San Francisco, CA), Copilot (Microsoft Corp., Washington, DC), Gemini (Google, Mountain View, CA), Meta AI (Meta, Menlo Park, CA), and Perplexity (Perplexity AI, Inc., San Francisco, CA).

Interaction with LLM chatbots

The interaction with LLM chatbots involved prompting each chatbot to generate a PLS from the abstract and title of each of the 30 selected studies. Two types of PLS were requested: structured and unstructured according to human-generated PLS type. The prompt instructed the chatbots to create PLS with - plain language, the reading level of sixth to eighth grade, length (as of human-generated PLS from PubMed), organizational structure (structured with subheadings or unstructured), active voice, and inclusive language. The template of the prompt was as follows:

Write a PLS of the article titled “X” from the following abstract “Y”. The PLS should be (continuous paragraph)/(structured with the following subheadings - introduction, methods, results, conclusion) written in plain language, with active voice, inclusive language, and readability suitable for sixth-eighth grade students.

The Centers for Disease Control and Prevention recommends that health education material should be written in such a way that it can be understood by anyone with eighth-standard education (USA standard) [15].

Readability

The readability of both human-generated and LLM-generated PLS was assessed using an online readability calculator (https://goodcalculators.com/flesch-kincaid-calculator). This tool provided quantitative measures of readability, including metrics such as the Flesch Reading (FR) Ease score, Flesch-Kincaid (FK) Grade Level, and other relevant indices. The calculator was previously used in other studies [16,17].

Quality assessment

The quality of the human-generated PLS and chatbot-generated PLS were checked by three assessors with predefined rating criteria on a 10-point scale with the following attributes - reporting major findings, ease of reading, ease of understanding, usage of active voice, usage of inclusive language, level of interpretation, accuracy of information. The questionnaire is available as Annexure 1 (Table 3). For the assessment, we first prepared a single printed sheet with a title, abstract, and PLS without writing any identification of the source (human or chatbot). A total of 30 such sheets were prepared for humans and six AI chatbots, yielding 210 sheets. The reverse page contained the rating scale. A total of three authors (HM, SM, PKS) rated the PLS and the average score of three rates was considered the final score for each attribute (a total of seven attributes). An overall score was also computed by taking the average score of the seven attributes.

Statistical analysis

Data were expressed in mean and standard deviation. The scores of PLS were compared by repeated-measure Analysis of Variance (ANOVA) with a post-hoc test, applicable if there is statistically significant variance. ANOVA was selected as it allows comparison across multiple chatbot outputs and human-generated PLS. Post-hoc tests identified significant differences between individual chatbots. We used GraphPad Prism 9.5.0 (GraphPad Software, Boston, MA) for statistical analysis. A p-value < 0.05 was considered statistically significant.

## Results

The LLM chatbots generated PLS with significantly lower FK grade levels (p-value < 0.0001), meaning the content was easier to understand for individuals with lower educational levels. Except for Copilot, all other LLM chatbots produced PLS with higher FR ease scores compared to human-written PLS, indicating that their content was generally easier to read and comprehend. Linguistic characteristics of the PLS are shown in Table 1.

**Table 1:** Linguistic characteristics of plain language summary by human and artificial intelligence-based chatbots All data are presented in mean ± standard deviation format. The p-values are of one-way analysis of variance (ANOVA) *Flesch-Kincaid (FK) Grade Level = 0.39 × (Total Words/Total Sentences) + 11.8 × (Total Syllables/Total Words) − 15.59. The higher the grade level, the easier to understand (if a grade level is 10, then a 10th-standard student can understand the text, USA standard) †Flesch Reading (FR) Ease Score = 206.835 − 1.015 × (Total Words/Total Sentences) − 84.6 × (Total Syllables/Total Words). The higher the ease score, the easier to read. ↑: Statistically significant higher value in post-hoc test in comparison to human-written plain language summary ↓: Statistically significant lower value in post-hoc test in comparison to human-written plain language summary

Parameter	Human	ChatGPT	Claude	Copilot	Gemini	Meta AI	Perplexity	P-value
FK grade level*	15.13 ^±^ 2.83	10.88 ± 1.76↓	9.73 ± 1.33↓	11.11 ± 2.89↓	7.39 ± 1.44↓	11 ± 1.88↓	12.85 ± 2.37↓	<0.0001
FR ease score†	22.57 ± 14.33	47.21 ± 8.94↑	54.54 ± 7.64↑	37.65 ± 20.38	68.75 ± 7.8↑	46.86 ± 11.64↑	38.13 ± 12.14↑	<0.0001
Average word per sentence	19.86 ± 4.8	16.95 ± 3.7	16.41 ± 2.75↓	12.38 ± 2.35↓	14.83 ± 2.85↓	17.27 ± 3	19.8 ± 3.88	<0.0001
Average syllables per word	1.95 ± 0.17	1.68 ± 0.1↓	1.6 ± 0.09↓	1.85 ± 0.25	1.45 ± 0.1↓	1.67 ± 0.15↓	1.76 ± 0.12↓	<0.0001
Sentences	7.53 ± 3.93	8.07 ± 5.39	8.43 ± 4.34	13 ± 5.91↑	6.63 ± 3.22	7.6 ± 4.45	7.63 ± 5.2	<0.0001
Words	147.4 ± 77.1	126.03 ± 59.22↓	132.63 ± 51.96	151.82 ± 50.76	94.67 ± 36.56↓	123.63 ± 45.8↓	138.93 ± 58.59	<0.0001

**Figure 2:**
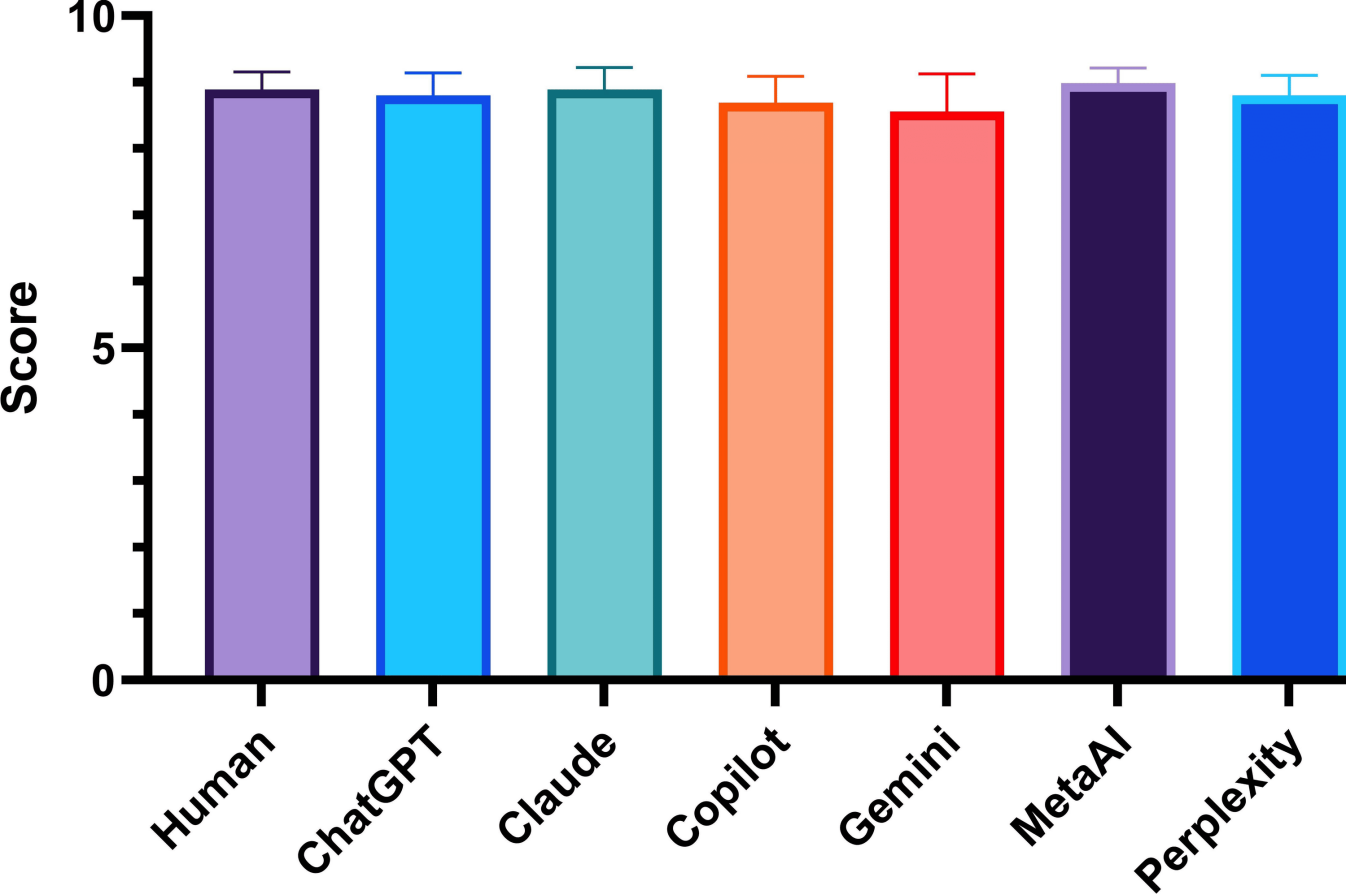
Overall score of plain language summary by human and artificial intelligence-based chatbots

The overall score of human-written PLS was 8.89±0.26 and although there was statistically significant variance among the scores (F = 7.16, p-value = 0.0012), in post-hoc test, there was no difference between human-generated and individual chatbots-generated PLS (ChatGPT 8.8±0.34, Claude 8.89±0.33, Copilot 8.69±0.4, Gemini 8.56±0.56, Meta AI 8.98±0.23, and Perplexity 8.8±0.3) and the comparative score is shown in Figure 2.

Gemini showed the lowest score in reporting major findings in PLS. Meta AI scored highest in ease of reading and understanding. All chatbots wrote in active voice and used inclusive language like humans did. Gemini showed the lowest score in the capability of interpretation of results. The accuracy of the chatbot-generated PLS is still lower than humans. Domain-wise scores of the PLS generated by the chatbots are shown in Table 2.

**Table 2:** Average (of three raters) score of plain language summary by human and artificial intelligence-based chatbots All data were presented in mean ± standard deviation format. The p-values are of one-way analysis of variance (ANOVA) ↑: Statistically significant higher value in post-hoc test in comparison to human-written plain language summary ↓: Statistically significant lower value in post-hoc test in comparison to human-written plain language summary

Item	Human	ChatGPT	Claude	Copilot	Gemini	Meta AI	Perplexity	P-values
Major findings	8.98 ± 0.42	8.78 ± 0.39	8.8 ± 0.44	8.6 ± 0.71	8 ± 0.35↓	9.02 ± 0.34	9.07 ± 0.51	<0.0001
Reading	8.76 ± 0.55	9.12 ± 0.36	9.22 ± 0.46	8.76 ± 0.65	9.29 ± 0.93	9.24 ± 0.29↑	8.86 ± 0.65	0.002
Understanding	8.8 ± 0.56	9.12 ± 0.36	9.16 ± 0.47	8.82 ± 0.51	9.27 ± 0.95	9.24 ± 0.29↑	8.82 ± 0.36	0.007
Active voice	8.72 ± 0.3	8.64 ± 0.53	8.87 ± 0.58	8.66 ± 0.53	8.71 ± 0.91	8.8 ± 0.37	8.54 ± 0.37	0.09
Inclusive language	8.76 ± 0.28	8.56 ± 0.49	8.59 ± 0.45	8.6 ± 0.54	8.49 ± 0.8	8.69 ± 0.28	8.57 ± 0.47	0.11
Interpretation	8.84 ± 0.46	8.41 ± 0.44	8.61 ± 0.48	8.5 ± 0.49	7.89 ± 0.52↓	8.74 ± 0.49	8.64 ± 0.49	<0.0001
Accuracy	9.4 ± 0.27	8.96 ± 0.34↓	8.97 ± 0.37↓	8.91 ± 0.55↓	8.22 ± 0.44↓	9.13 ± 0.31↓	9.12 ± 0.44↓	<0.0001

## Discussion

In this study, we found that the LLM chatbots can generate PLS with lower FK grade levels, indicating simpler language. Most chatbots, except for Copilot, produced PLS with higher FR Ease scores than human-written summaries. The accuracy of chatbot-generated PLS still slightly lags behind that of human-written summaries.

The rise in articles featuring PLS over the years reflects an increasing emphasis on making research more accessible to a broader audience. An author should write PLS according to reading age and literacy level based on the intended audience [18]. This is suitable for the patient’s educational material. However, the scientific article is published online and on print media and can be accessed by a population with variable degrees of education. Hence, it is better to keep it as simple as it can be [15]. The lower FK grade levels achieved by LLM chatbots suggest that these AI models are proficient in generating simpler, more digestible content [19]. The higher FR Ease scores, except for those generated by Copilot, further emphasize the chatbots' ability to produce more readable text, which is crucial for PLS.

The absence of significant differences between human-written and chatbot-generated PLS scores indicates that these AI models are approaching human-level proficiency in writing summaries. However, the overall lower accuracy of chatbot-generated PLS underscores the challenges these models may face in fully conveying scientific content. Hence, human oversight remains essential to ensure accuracy and completeness [20].

Van Veen et al. demonstrated that LLMs specifically tuned for medical science can outperform humans in text summarization [11]. While we found that the accuracy of PLS is lower, the overall quality is similar to human-written PLS. We used general-purpose LLMs that are available free, and Van Veen et al. used tuned LLM. Tang et al. found that general-purpose LLMs, such as ChatGPT, can summarize text but often produce inconsistent summaries [12]. Our findings do not fully support this; however, we observed that the accuracy of the content is lower than human-generated PLS. This necessitates the need for caution when relying on general-purpose LLMs for precise scientific communication. Ovelman et al. reported that Claude 2 can generate PLS with accuracy and only minor errors [13]. Our study aligns with this and in the overall score chatbot-generated PLS is as good as human-written PLS. As the field of AI is evolving, there is a further need for research and evaluation of the capability of LLM in generating PLS.

The findings of this study have significant implications for the scientific community in India, where language diversity and varying levels of English proficiency can pose challenges for effective research dissemination. By using LLM chatbots, researchers can overcome language barriers and improve the clarity and accessibility of their work [21]. This not only fosters greater public engagement with science but also contributes to the overall advancement of knowledge and innovation in the country [22].

However, there may be some limitations to using chatbots in writing PLS. Using any machine to generate text has the potential to kill the creativity and writing capability of humans and they can be more dependent on machines in the future [23]. In addition, there are guidelines set by the International Committee of Medical Journal Editors [24] and the World Association of Medical Editors for using LLM chatbots in the writing process [25]. Authors should acknowledge the details of the tool along with the nature and extent of using chatbots in the writing process for ethics and transparency. In addition, AI can hallucinate or make mistakes in rare instances. Hence, there should always be human oversight.

This study has several limitations. The sample size of 30 articles may not fully capture the variability in the quality and style of human-generated PLS. Additionally, the selection of six LLM chatbots, based on current literature and author consensus, may not represent the full spectrum of available AI tools. Moreover, the study focuses solely on English-language summaries, which may not address challenges related to generating PLS in other languages. A limitation of the cross-sectional design is that it does not account for temporal changes in chatbot capabilities or improvements in human writing skills over time. While Quillbot AI content detector was used, AI detection tools are not foolproof and may misclassify human-generated content as AI-generated and vice versa. Future studies could validate findings using multiple AI detectors. Chatbot outputs can vary slightly across multiple runs. To minimize variability, each chatbot-generated PLS was run once per abstract. Future studies could assess output consistency by generating multiple versions and comparing them. In addition, a longitudinal study could track the evolution of chatbot-generated PLS quality over multiple years as AI models improve.

## Conclusions

This study demonstrates the potential of LLM chatbots in generating PLS from scientific abstracts, highlighting their capability to produce accessible and clear summaries. Hence, LLM chatbots can aid non-native English speakers by overcoming language barriers and improving the readability of their work. This help from LLMs would benefit individual researchers and contribute to a more inclusive and comprehensible dissemination of scientific knowledge to a broader audience. While LLM chatbots improve readability, they may introduce minor inaccuracies also. Hence, PLS generated by LLM should always checked for accuracy and relevancy.

## Appendices

Appendix 1

**Table 3:** Questionnaire for rating the quality of plain language summary

Item	Rating scale									
	1	2	3	4	5	6	7	8	9	10
1. Reporting major findings										
2. Ease of reading										
3. Ease of understanding										
4. Usage of active voice										
5. Usage of inclusive language										
6. Level of interpretation										
7. Accuracy of information										
8. Overall score calculated ((sum 1 to 7)/7)										
